# “We follow what our elders taught us”: an ethnographic study of kinship, rituals, and reproductive transitions among women in the GaRBH cohort, Rajasthan, India

**DOI:** 10.3389/fgwh.2026.1915333

**Published:** 2026-09-30

**Authors:** Sonia Maurya, Gagandeep Kaur Walia, Subhash Kumar Jha, Tripti Agarwal, Ruchi Saxena, Helen Lambert, Vipin Gupta

**Affiliations:** 1 Public Health & Genomics Labs, Department of Anthropology, University of Delhi, Delhi, India; 2 CHARD, TSB, Ashoka University, Sonipat, India; 3 Environmental Health, Centre for Chronic Disease Control, New Delhi, India; 4 Department of Obstetrics and Gynaecology, Sardar Patel Medical College, Bikaner, India; 5 Department of Population Health Sciences, Bristol Medical School, University of Bristol, Bristol, United Kingdom

**Keywords:** ethnographic research, India, infant care, postpartum cultural practices, pregnancy rituals, Rajasthan, women's health

## Abstract

This paper examines the cultural meanings and ritual practices surrounding pregnancy, childbirth, and early infant care in Bikaner, and their implications for maternal and newborn wellbeing. We conducted a qualitative ethnographic study based on in-depth interviews and short-term observations of household rituals with 33 participants, including 13 pregnant women, 11 recently delivered mothers, and 9 elder female family members. The findings reveal that pregnancy is widely perceived as a vulnerable and transformative phase that necessitates heightened care, protection, and collective familial support. Rituals such as *godbharai, chathiar,* and *annaprashan* are ceremonial practices that function as inherent mechanisms through which families express care, strengthen their kinship ties, and provide emotional reassurance to women during periods of bodily and social transition. Similarly, beliefs associated with the placenta, maternal blood, and postpartum seclusion further illuminate how women and families interpret and make sense of reproductive experiences through local cultural frameworks. At the same time, the study also identifies some long-held customs, particularly restrictive dietary norms, postpartum restrictions, and delays in seeking biomedical care, that may adversely affect maternal nutrition and neonatal health outcomes. Rather than framing tradition and biomedicine as oppositional, the paper argues for a culturally sensitive public health approach that recognizes the social and emotional significance of ritual practices while promoting safe and evidence-based maternal and newborn care.

## 1 Introduction

Pregnancy is the state of a woman or individual after conception (fertilization of an egg by a sperm) until birth, during which a developing human (fetus) grows inside the uterus (1). It is frequently discussed through the language of health indicators, risk reduction, institutional delivery, and maternal care utilization. However, pregnancy is more than a biological event; it is often experienced by women and families simultaneously as a social, emotional, symbolic, and relational process and event with a range of social consequences. Across societies, pregnancy often reorganizes households by redistributing labour, transforming kinship obligations and creating new social identities. Since pregnancy comes with many challenges and uncertainties, it also produces ritual practices that seek to manage the uncertainties surrounding reproduction.

Globally, communities observe a wide range of practices across pregnancy and childbirth, including food restrictions, blessing ceremonies, prepartum confinement practices, postpartum confinement, newborn purification rituals, infant protection practices, and naming ceremonies that accompany the incorporation of the newborn into social life (2, 3). Practices which have been widely observed and reported from across the globe are beliefs and practices associated with the placenta, which is a temporary organ that develops during pregnancy to connect the fetus with the mother for nutrient, oxygen, hormone, and waste exchange, and becomes functionally redundant after childbirth when it is expelled from the body. In many Indigenous communities, the placenta is regarded as sacred and imbued with spiritual and social significance for both mother and child, giving rise to two major type of practices which include burial of placenta within household, planting trees at burial sites and in rare cases placental consumption aimed at promoting maternal recovery, fertility, and wellbeing (3, 4). Studies have further documented these rituals are intended to protect the newborn from evil spirits, ensure future wellbeing, and support the social incorporation of the mother-child dyad within the community (2).

Along with such beliefs and practices, some communities consume the placenta following childbirth, a practice commonly referred to as placentophagy. Studies from across the globe have documented practices of placentophagy in which the placenta is consumed raw, cooked, dried, powdered, or encapsulated, particularly among communities where it is believed to promote maternal recovery, enhance lactation, reduce postpartum bleeding, restore fertility, and prevent postpartum depression (3, 5). Placentophagy, in some communities, is also viewed as a symbolic and therapeutic practice associated with healing, bodily restoration, and the transition into motherhood. These practices also illustrate how the placenta acquires multiple and shifting identities as waste, therapeutic tool and symbol of entanglement or ritual object, offering insight into negotiations of health, agency and trust in contemporary obstetric care (6). Conversely, biomedical literature has raised concerns regarding infection risks and limited scientific evidence supporting some of placentophagy's claimed benefits (7, 8).

In south Asian countries, especially in India, communities observe a range of rituals and practices associated with pregnancy and birth. Studies among North Indian communities have documented rituals such as Godh Bharai ceremonies during pregnancy, postpartum cleansing rituals, naming ceremonies, newborn hair removal rituals, and customs surrounding placental disposal (9, 10). Other commonly reported practices include using old-generation clothes for newborns, applying kajal (lampblack) to protect infants from the evil eye, keeping knives or water near the mother and baby for spiritual protection, delaying newborn bathing, giving honey or sugar before breastfeeding, and the use of protective amulets or taweez (11, 12).

While many of these practices continue to provide emotional support and family involvement during the perinatal period, some beliefs may also intersect with biomedical concerns related to breastfeeding, newborn care, infection prevention, and maternal health practices. At the same time, increasing attention has been given to the importance of culturally sensitive maternity care that respects women's beliefs and ritual practices while ensuring safe maternal and newborn health services (3, 13).

Studies from some pastoral communities in rural Rajasthan have documented practices such as placental burial, special postpartum feeding, and protective care rituals following goat delivery, reflecting how reproductive transitions in animals are also culturally managed through indigenous knowledge systems. Women rely on traditional knowledge to identify pregnancy and labor signs, provide care during gestation, and manage kidding and neonatal care. A commonly reported postpartum practice is the burial of the placenta after delivery to prevent it from animal consumption and to maintain hygiene and social norms (14).

With expansion of institutional delivery, antenatal care, and maternal health programs, pregnancy has increasingly become a biomedical event. Such expansions have substantially transformed how women experience pregnancy and childbirth. Yet, despite advances in obstetric care and expanding institutional childbirth, pregnancy continues to involve risks that remain emotionally, socially, and existentially difficult to manage. Rather than disappearing with modernization, many socio-cultural practices surrounding pregnancy continue to persist, adapt, and coexist alongside biomedical systems. Pregnancy today is produced through the coexistence of biomedical institutions and ritual systems. Medicalization has changed where pregnancy occurs, who manages it, and how it is experienced, but there are many symbolic practices that still continue.

This paper traces and maps some of these symbolic practices including rituals, practices, and associated beliefs surrounding pregnancy and childbirth and examines how they function within vernacular communities to organize reproductive experiences, social relations, and transitions into motherhood. It further examines how these rituals and practices are being transformed under conditions of increasing medicalization. Drawing from ethnographic observations from fieldwork conducted as part of a cohort study in Bikaner district in Rajasthan, India, the paper argues that while medicalization has altered the location, actors, and performance of reproductive practices, it has not eliminated their social functions. Instead, pregnancy increasingly exists simultaneously within two domains: the biomedical world of institutional care and the social world of kinship, ritual, and symbolic meaning. This paper specifically deal with the second domain.

## 2 Methodology

### 2.1 Study site and cohort description

This study was part of a larger prospective pregnancy and birth cohort study, titled GaRBH cohort (Gestational Route to Healthy Birth), that was established between 2018 and 2023 in Bikaner District, Rajasthan, India. The GaRBH cohort is the first systematically planned longitudinal study in Indian anthropology. It was located at the largest public hospital in the district, a tertiary healthcare facility with over 1,700 beds that serves a population of about three million people across Bikaner and the surrounding districts of Ganga Nagar, Hanuman Garh, Churu, and Sikar. Broadly, this cohort had the purpose of investigating metabolic changes during pregnancy as related to gestational weight gain and included systematic monitoring of antenatal mental health, nutrition, and physiological status during pregnancy (15). Pregnant women were included in the cohort through predefined inclusion and exclusion criteria and systematically followed three times up to delivery after recruitment at baseline during the first trimester. During observation, delivery and postnatal care follow-ups, women and their families often requested to keep the placenta, and sometimes placental tissues and umbilical cord, while also sharing the cultural beliefs and practices associated with it. This led to the further exploration of practices associated with pregnancy and postnatal care. Data were collected during the women's stay in the postnatal care wards and also during postnatal follow-ups at their households, which were mostly done for observing the growth of the infant. In some cases, families invited research team members to attend pre- and post-delivery feasts and ceremonial occasions. This also facilitated in-person observation of these rituals and enabled understanding their significance through formal and informal interactions with family members, particularly elderly women of the family.

### 2.2 Sampling and participants

The study adopted a purposive sampling strategy. Participants included pregnant women and recently delivered mothers enrolled in the GaRBH cohort, along with elderly female family members (primarily mothers-in-law and grandmothers) accompanying these participants. Selection for qualitative interviews was based on participants' willingness to discuss their experiences and cultural practices related to pregnancy, childbirth, and the postpartum period, as well as opportunities to conduct participant observation during hospital and household follow-up visits. As participants were recruited from the ongoing GaRBH cohort, refusal to participate in the qualitative study was minimal. However, some eligible participants could not be included because they were unavailable during the data collection period, primarily due to temporary relocation to their maternal home after childbirth, missed follow-up visits, or other logistical constraints. Eligible participants included pregnant women and recently delivered mothers enrolled in the GaRBH cohort, as well as elderly female family members accompanying these participants who possessed experiential knowledge of pregnancy, childbirth, and postpartum rituals. Pregnant women at different gestational stages and with varying parity were purposively included to capture diverse reproductive experiences. Participants were required to be aged 18 years or above, able to communicate in Hindi or Marwari, and willing to provide written informed consent. Women who declined participation or were unable to provide informed consent were excluded from the study. No eligibility criteria based on maternal health status or pregnancy complications were applied, as the study aimed to explore sociocultural experiences rather than clinical outcomes. In total, 33 females participated, who included pregnant women (*N* = 13), postpartum mothers (*N* = 11), and elderly female family members (*N* = 9) accompanying the participant. Their ages ranged from 22 to 78 years, thus allowing for an intergenerational understanding of how ritual knowledge and practices are reproduced and transmitted across time. The participant flow in the current study is shown in Figure 1.

**Figure 1:**
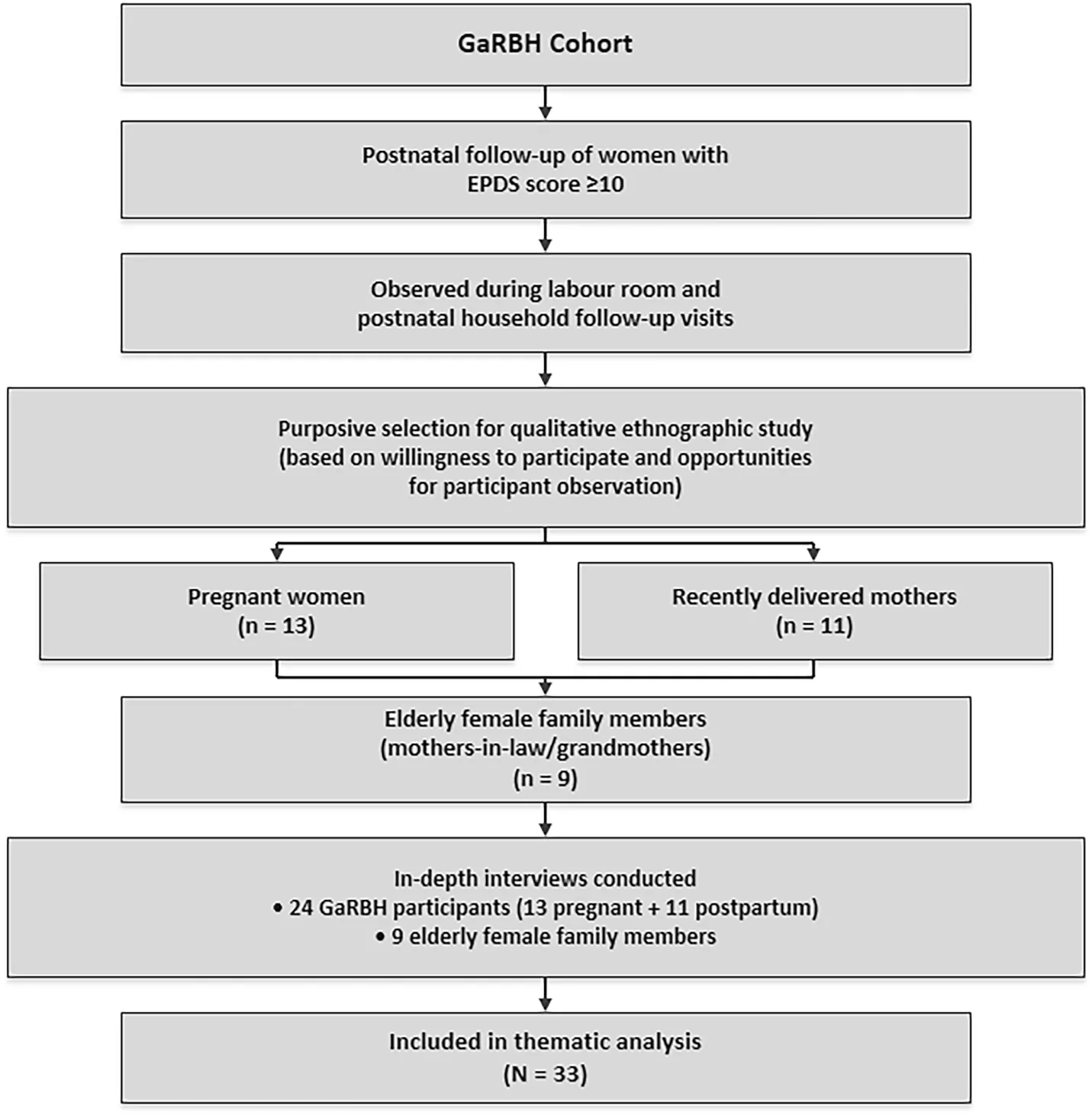
Participant flow diagram.

### 2.3 Study design and data collection

A qualitative ethnographic design was adopted to explore how pregnancy, childbirth, and early infant care are experienced and understood by women and their families in their sociocultural context. Fieldwork was conducted between 2023 and 2025 by a trained qualitative researcher. Data collection comprised a combination of in-depth interviews (IDIs) and participant observations. Short-term observations of ritual events such as *Godbharai, Chathiar* and *Annaprashan* were made in household settings, with detailed field notes capturing ritual sequences, gestures, material objects and the emotional atmosphere surrounding these events. In addition, observations during intrapartum and postpartum periods documented real-time practices associated with childbirth. All interviews were conducted in Hindi or Marwari in participants' homes and typically lasted between 45 and 90 min. All interviews were transcribed verbatim in the original language (Hindi or Marwari) and translated into English by the first author, who is fluent in both languages. To ensure that the meaning of participants’ narratives was accurately retained, translated transcripts were carefully reviewed alongside the original transcripts during data analysis. Culturally specific terms, such as Godbharai and Chathiar, were retained in their original language where no direct English equivalent existed. Their meanings were explained when first introduced in the manuscript to preserve their cultural context. A semi-structured interview guide explored pregnancy experiences, meanings attributed to rituals, postpartum care and support. Interviews were audio-recorded with consent from participants, transcribed verbatim and subsequently translated into English. Field notes, analytical memos and observation summaries were prepared side by side throughout data collection process, together with reflexive notes to document positionality, evolving relationships with participants, and the potential influence of the researcher's presence on the research process. Regular peer debriefing supported analytical reflexivity and enhanced interpretive rigor.

#### 2.3.1 Researcher positionality and reflexivity

The primary fieldwork was conducted by the first author, a female researcher with a Master's degree in Anthropology and prior experience in maternal and child health research. Although she was not a member of the local community, being from the neighbouring state of Haryana facilitated cultural familiarity while maintaining sufficient analytical distance during data collection and interpretation. Her gender facilitated rapport and open discussions with pregnant women, recently delivered mothers, and elderly female family members on culturally sensitive issues related to pregnancy, childbirth, and postpartum practices. Throughout the fieldwork, reflexive memos were maintained to document the researcher's assumptions, interactions with participants, and evolving interpretations of the data. Regular peer debriefing with senior members of the research team was undertaken to critically examine emerging findings, discuss alternative interpretations, and minimize the influence of potential researcher bias, thereby enhancing the credibility and trustworthiness of the analysis.

### 2.4 Data analysis

Data analysis integrated inductive thematic coding within interpretive and symbolic anthropological frames. Transcripts, field notes, and observation summaries were entered into NVivo to support systematic and iterative coding. Initial open coding focused on the identification of recurrent ideas related to purity, protection, kinship, and embodied experience. Axial coding then explored relationships among these categories, and selective coding synthesized central ritual logics and underlying cultural meanings. Interpretive analysis drew on symbolic anthropology to examine how practices of feeding, cleansing, burying, or applying substances create and express moral, and social significance in the local context. Analytical rigor was enhanced through peer debriefing and independent coding by a second analyst. Reflexive memos were kept throughout the analysis, continuously reassessing the researcher's assumptions and interpretive positions. The study followed Lincoln and Guba's criteria for qualitative rigour, ensured through prolonged engagement, triangulation across interviews, observations, informal member checking and ongoing peer consultation (16).

The first author coded all interview transcripts and field notes. The codes and emerging themes were regularly discussed with senior members of the research team through peer debriefing to improve the consistency and credibility of the analysis. The analysis combined an inductive approach, where codes were developed from the data, with a deductive approach guided by the study's theoretical framework. Similar codes were grouped into categories, which were then refined into broader themes. Data collection and analysis continued simultaneously until no new themes or insights emerged from the interviews and observations, indicating that thematic saturation had been reached. This study followed the Consolidated Criteria for Reporting Qualitative Research (COREQ) checklist; the completed checklist is provided in Supplementary Table S1.2.

### 2.5 Theoretical framework

This paper draws on three complementary theoretical perspectives: rites of passage theory (17), the concept of liminality (18), and anthropological scholarship on kinship and social reproduction (19, 20). Together, these perspectives provide a framework for understanding how ritual practices surrounding pregnancy, childbirth, and the postpartum period organize reproductive transitions and shape social relationships.

Van Gennep (1909) argued that major life transitions are not merely individual experiences but socially organized processes that require ritual recognition and management (17). According to his theory of rites of passage, transitions between social statuses are structured through three phases: separation, liminality, and incorporation. During the separation phase, individuals move away from their previous social position; the liminal phase represents an intermediate period characterized by transition and uncertainty; and incorporation marks entry into a new social status (17). Pregnancy and childbirth closely reflect this sequence. Pregnancy-related restrictions, ritual observances, and preparations may be understood as forms of separation from ordinary routines, while childbirth and the postpartum period represent transitional phases through which women move toward socially recognized motherhood. Subsequent rituals facilitate the incorporation of both mother and child into family and community life.

Building upon Van Gennep's work, Turner (1969) conceptualized liminality as a condition of ambiguity in which individuals occupy a position between established social categories. Liminal periods are often associated with uncertainty, vulnerability, and heightened attention to ritual activity. Pregnancy and childbirth can be understood as liminal states because women undergo profound physical, emotional, and social transformations while occupying an intermediate position between previous and future identities. Similarly, newborns may be viewed as existing in a transitional state prior to their full incorporation into social life. Rituals performed during pregnancy, childbirth, and the postpartum period can therefore be understood as mechanisms that manage uncertainty, provide protection, establish social recognition, and facilitate movement through transitional stages (17, 18).

While rites of passage and liminality explain the structure of reproductive transitions, scholarship on kinship and social reproduction helps explain how these transitions are collectively organized and sustained within families. Social reproduction refers to the processes through which social relationships, cultural values, knowledge, and identities are reproduced across generations. From this perspective, reproduction extends beyond biological birth to include the social production of persons, relationships, and family continuity. Pregnancy and childbirth therefore represent not only biological events but also important moments through which kinship ties are reaffirmed, lineage continuity is maintained, and cultural knowledge is transmitted (17–20).

Anthropological studies have long demonstrated that reproductive experiences are embedded within kinship systems and household relations. In many South Asian contexts, pregnancy and childbirth are collective family events involving mothers-in-law, grandmothers, sisters-in-law, and other female relatives who play central roles in guiding, supporting, and monitoring women throughout the reproductive process. Through ritual participation and caregiving practices, elder women transmit reproductive knowledge, reinforce social norms, and facilitate the incorporation of mothers and newborns into existing kinship structures. Rituals associated with pregnancy, childbirth, and infant care therefore function not only as symbolic practices but also as mechanisms through which family relationships and social obligations are reproduced across generations (20).

Previous scholarship has frequently used rites of passage and liminality frameworks to examine childbirth and transitions into motherhood. However, less attention has been paid to how multiple ritual practices operating across pregnancy, childbirth, and the postpartum period collectively structure reproductive transitions while simultaneously reproducing kinship relations and family continuity. By integrating rites of passage theory, liminality, and social reproduction, this paper conceptualizes pregnancy rituals as interconnected practices through which women and newborns are protected, socially recognized, and incorporated into family and community life. This framework guides the analysis of how ritual practices in Rajasthan organize reproductive transitions, manage uncertainty, and sustain kinship relations across generations (Figure 2).

**Figure 2:**
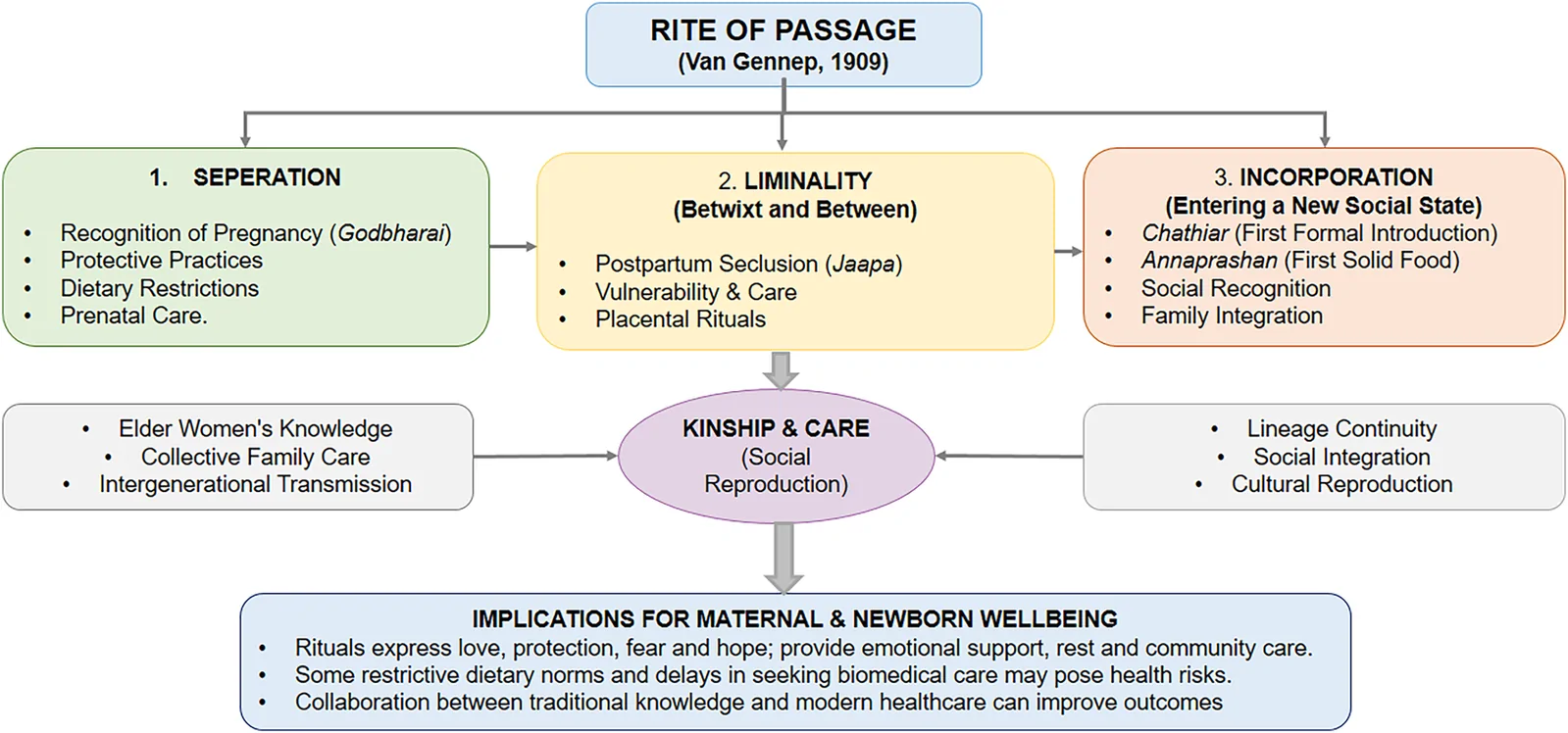
Pregnancy and childbirth as rites of passage: a conceptual framework. The framework shows how pregnancy and childbirth rituals in Bikaner operate as a process of separation, liminality and incorporation, supported by kinship, beliefs and collective care, resulting in the social reproduction of individuals and lineages.

## 3 Results

We conducted a total of 33 IDIs, among which 24 were with the women who were pregnant at the time of interview (*N* = 13) or had recently delivered and were new mothers (*N* = 11). Along with these 5, elderly female relatives were also interviewed. The interviews captured a wide range of experiences related to pregnancy, childbirth, and newborn care, and five major themes were identified that reflected the cultural logic, ritual practices, and support systems in shaping pregnancy and childbirth experiences in Bikaner. These five themes included the meaning of the placenta; pregnancy celebrations and preparation rituals; food, purity, and early nourishment; postpartum confinement and care from women kin; and infant cleansing and protection rituals. Among the 28 women interviewed for their lived experience, mostly were in their second trimester (62.5%), with smaller proportions in the first (12.5%) and third trimesters (25%). Age of respondents ranged between 18 and 30 years, with a mean age of 24.12 years (SD ± 3.57). Nearly half of the participants (45.8%) were primiparous, while others had experienced two (16.7%) or three or more pregnancies (37.5%). Only a small proportion of women (3.7%) had not completed secondary education, while the majority had completed secondary (50%) or senior secondary education (29.1%). Most households were in the lower-income range, with 20.8% reporting a monthly income below INR 10,000. Most participants lived in joint and extended family households (79.1%), while a smaller proportion lived in nuclear families (20.8%). These characteristics reflected a predominantly young, semi-educated population navigating pregnancy within extended family settings (Table 1).

**Table 1:** Socio-demographic characteristics of study population.

Variables	*N* = 24
	Count (%)
Age (years)	
Mean (±SD)	24.12 (3.57)
18–23	11 (25.8)
≥24	
Gravida	13 (54.1)
Primiparous	11 (45.8)
2nd	4 (16.7)
≥3rd	
Education	9 (37.5)
Primary school	1 (4.1)
Secondary school	12 (50)
Senior secondary school	7 (29.1)
Graduation	3 (12.5)
Post-graduation	
Household income	1 (4.1)
<10,000	5 (20.8)
10,001–30,000	12 (50)
>30,001	5 (20.8)
Don't know	2 (8.4)
Family structure	
Nuclear	5 (20.8)
Joint & extended	19 (79.1)

This study reflects the region's predominantly patrilineal and virilocal social structure, in which women typically marry outside their natal village and reside with their husband's family after marriage. Within these households, daughters-in-law may occupy a relatively subordinate position, while elder women, particularly mothers-in-law, play an important role in decision-making related to pregnancy, childbirth, and childcare. The intergenerational role of elder women also reflects processes of social reproduction, through which cultural knowledge, ritual practices, and norms surrounding pregnancy and childbirth are transmitted and sustained across generations within the household. Practices such as veiling and gendered norms of modesty were observed in some households and influenced women's mobility and interactions. The study cohort comprised predominantly Hindu participants. The findings were organized into three phases i.e., separation, liminality, and incorporation, to illustrate how women's reproductive experiences and associated ritual practices unfolded across pregnancy, childbirth, the postpartum period, and early infancy. The separation section focuses on pregnancy celebrations and preparation rituals, including *Mehendi* and *Godbharai*, which participants described in relation to the recognition of pregnancy, kinship support, and preparation for motherhood. The liminality section encompasses practices associated with childbirth and the postpartum period, including placental handling, dietary and food-related practices, purification rituals, restricted movement, and protective care. The incorporation section focuses on infant cleansing and protection practices, *Chathiar*, and *Annaparashan*, which participants described in relation to the newborn's gradual inclusion within household rituals, relationships, and social life. Across these phases, the involvement of mothers-in-law, grandmothers, and other elder female relatives highlighted the intergenerational transmission of ritual knowledge and practices. The thematic distribution of the findings and observations is presented in Table 2, while the detailed thematic analysis and verbatim quotations are provided in the Supplementary Table S1.1.

**Table 2:** Major themes and topics emerging from the thematic analysis.

Sections	Themes	Sub-themes
Separation	Pregnancy Celebrations and Preparation Rituals	*‘Mehendi’* for Comfort and Blessing
		*‘Godbharai’* as Collective Celebration
		Gifts as Expressions of Care and Support
Liminality	Symbolic Meaning and Beliefs Associated with the Placenta	Placental Burial Practice within Household Space
		Ritual Handling ‘*Aawal’* After Birth
	Food, Purity, and Early Nourishment	Dietary Practices and Activity Restrictions during Pregnancy
		*‘Bhog Bharai’* and Sacred Food Preparation
		Ritual Rules about Keeping Food within the Home
		Purifying the Mother's Milk and First Feeding Practices
	Postpartum Care and Support	Rest and Restricted Movement
		Protective Rituals
Incorporation	Infant Cleansing and Protection Rituals	Dough-Based Cleansing
		*‘Chathiar’* Purification ceremony
		Use Holy Water of River Ganga for purification
	Food and Early Nourishment	*‘Annaparashan’* Introducing First Solid Food

Section I: Separation.

### 3.1 Pregnancy celebrations and preparation rituals

Across interviews, pregnancy emerged not merely as a biological process but as the beginning of a socially recognized transition into motherhood. Through rituals such as *Mehendi, Godbharai,* and ceremonial gift exchange, families publicly acknowledged the expectant mother's changing social identity while preparing her emotionally, socially, and symbolically for motherhood. These practices collectively marked the first stage of the reproductive transition, where kinship networks actively participated in recognizing, supporting, and legitimizing pregnancy within the community.

#### 3.1.1 ‘*Mehendi’* for comfort and blessing

Participants described the application of *mehendi* (henna) to the hands and feet as an important ritual during pregnancy, typically performed during the Godbharai ceremony by female relatives, particularly elder women in the household. *Mehendi* was viewed as both soothing and auspicious for the pregnant women. It was explained that the cooling effect of *mehendi* provided physical comfort, while its presence signified happiness within the household. One participant expressed,

When I get mehendi applied on my hands, I feel a kind of calm. It brings a cooling sensation in the body. It feels like a happy time in the household.

For many, mehendi marked the beginning of celebration during pregnancy and served as a visual expression of collective joy.

#### 3.1.2 ‘*Godbharai’* as collective celebration

The *Godbharai* (“filling the lap”) ceremony was described as a central event during pregnancy, typically celebrated in the late second or third trimester, once the pregnancy becomes visibly apparent and is socially acknowledged within the community. It marks an important moment when the woman's pregnancy is publicly recognized and celebrated.

The ceremony involved the participation of close kin, particularly women from both the natal and marital families, including mother, mother-in-law, sisters, sisters-in-law *(nanad)*, and other female relatives and neighbours. These participants gathered to “fill the mother's lap” with grains, sweets, coconut, and turmeric-coloured cloth, symbolizing blessings for abundance, a safe pregnancy, and the well-being of the child. As one participant explained:

During Godbharai, everyone gathers to fill the lap with grains, sweets, coconut, and yellow cloth. While giving these things, they say it is good for the mother and child. And truly, on that day, it feels like everyone is with me, everyone is giving their blessings.

Participants noted that the event brought together women across generations, creating a shared space for songs, encouragement, and the transmission of pregnancy-related knowledge. Particular importance was attached to the presence of the husband's sister *(nanad),* who, even after marriage, often returned from her marital home to participate in the ceremony and perform specific ritual roles. These included assisting in the *god bharna* ritual by placing symbolic items such as grains, sweets, and cloth in the expectant mother's lap, leading or participating in ritual songs, and playfully engaging with the expectant mother in ways that helped ease anxiety and mark the celebratory nature of the occasion. In some instances, she also acted as an intermediary between the natal and marital families, facilitating the exchange of gifts and maintaining ritual continuity. Such participation reinforced kinship ties across households and highlighted the continued involvement of natal and affinal relations in reproductive rituals. Participants also described these interactions as occasions through which younger women learned ritual practices and expectations surrounding pregnancy from mothers, mothers-in-law, sisters, and other elder female relatives.

When a ritual takes place, there is a warmth in the house. My mother-in-law, sister-in-law, everyone speaks more gently. At that time I feel that pregnancy is not only pain and difficulty… there is joy too.

These practices not only affirmed the social recognition of pregnancy but also fostered emotional reassurance and a sense of belonging for the expectant mother. Participants' accounts therefore described Godbharai as both a celebration of pregnancy and a collective preparation for the woman's transition towards motherhood.

#### 3.1.3 Gifts as expressions of care and support

Gifts offered during Godbharai includes the food items, clothing, and household produce that were described as expressions of blessings for abundance in life along with affection and concern. These offerings made the expectant mother feel cared for and socially included. A few participants who were not in a situation where these rituals could be performed, expressed their dissatisfaction and feelings of being relatively less valued. These were particularly those who were facing domestic violence or living alone in nuclear families due to economic reasons or household conflicts. A younger participant described the atmosphere of the ceremony:

On the day of Godbharai, all the women sing together, laugh, talk….sometimes jokes, sometimes old stories. In those moments I feel, ‘yes, I’m not alone.’ But… (pause) sometimes it hurts too, like when there is conflict at home or no one to support me. But during these rituals, the heart feels held.

Women consistently emphasized that these gatherings strengthened relationships within the family and emphasized their value in the marital household during pregnancy. Women expressed feelings of safety and care through these rituals that were absent when not performed:

My Godbharai never happened… who would do it? It's just me and my husband here, and he is out working all day. Sometimes I feel… (long pause) maybe I'm not important to anyone. When I see videos of other women whose whole families gather, I feel emotional.

Section II: Liminality.

### 3.2 Symbolic meaning and beliefs associated with the placenta

Across our interviews, we found that the placenta *(“Aawal”)* was regarded as a significant and respected part of childbirth. Participants consistently described it not as biological waste but as an extension of the newborn, carrying the significance of first major layer of protection for the child and spiritual value. Many women were of the opinion that the placenta had remained with the child throughout pregnancy, so it should continue to be treated with care after birth. As one elder participant noted,

The placenta is not just a part. We call it the ‘first guardian,’ and that's what it is. As long as the baby is in the womb, it protects. Once a hospital person asked me if they should throw it away. I immediately said, ‘No, it stays with the baby, keep it safe…these ritual we have learned from our parents..

This understanding was shared across generations, with several participants referring to the placenta as the baby's “shield” or “first companion”. The belief that the placenta absorbed nourishment and influences during pregnancy shaped the care taken in its handling. One mother explained,

“It stays with the baby throughout, so it must be taken care of properly.”

Participants also described clear and deliberate practices regarding the disposal of the placenta. Burial of placenta *(“Aawal”)* within the household boundary was the most common practice. Many families chose the kitchen or a sheltered corner of the courtyard, spaces that are considered pure in the sense of nourishment/fertility and central to the home's wellbeing. One woman shared,

We always bury it in the corner of the kitchen. That place… I mean, it is the most sacred. When I became a mother for the first time, my mother-in-law held my hand and showed me where they bury it, saying it keeps the baby's good fortune inside the home.

Some families emphasized directional placement, such as burying the placenta in a north–south alignment to promote the newborn's safety and growth. Keeping the placenta within the home compound was described as a way of ensuring that “the child's good fortune remains inside.”

Although placental burial was common, families also described additional steps taken before the placenta was laid to rest. Some washed and dried it, viewing this as the completion of the birth process. In a few households, a small portion of the placenta was preserved as a protective charm. As one mother explained,

Sometimes we wrap a small portion in silver. Yes… just a little bit. My mother-in-law would say, ‘Newborns attract the evil eye, this protects them.’ I have two children, and I still have the pieces for both.

Across narratives, these practices were described as the newborn's first interaction with the household's rituals. Participants expressed that caring for the placenta, whether through washing, wrapping, burial, or preservation, represented the symbolic act of welcoming the baby into the family's social and domestic space. Participants' descriptions of the placenta also linked childbirth with the baby's changing relationship to the household. Practices such as washing, wrapping, preserving, and burying the placenta were described as ways of completing the birth process and ensuring the child's protection and good fortune within the family.

### 3.3 Food, purity, and early nourishment

Participants described food-related rituals as central to both pregnancy and early postpartum care. These practices were closely connected to ideas of purity, protection, and the wellbeing of the mother and newborn. Women also reported following specific dietary practices and activity restrictions during pregnancy, which were considered important for maintaining maternal health and safeguarding the unborn child. Families reported that the preparation, and consumption of specific foods carried symbolic meaning and played an important role in early nourishment.

#### 3.3.1 Dietary practices and activity restrictions during pregnancy

Participants described pregnancy as a “delicate time” requiring careful attention to diet and daily activities in order to protect the mother and unborn child. Many women reported following specific food-related practices based on culturally defined classifications of “hot” and “cold” foods. Foods such as curd, papaya, and pineapple were often avoided, as they were believed to cause weakness or increase the risk of miscarriage. Similarly, heavy or oily foods were restricted due to concerns about digestion. At the same time, women described consuming foods considered strengthening, including ghee, jaggery, dry fruits, milk, and panjiri, which were believed to support maternal health and promote fetal growth.

One has to be very careful… you can't eat everything. Foods like curd, papaya, and pineapple are considered ‘cold,’ so they are avoided. My mother-in-law would say these could make the baby weak. So I mostly consumed ghee, milk, and panjiri to maintain strength.

In addition to dietary practices, participants also reported avoiding certain activities during pregnancy, such as lifting heavy objects, walking long distances, going out alone after dark, or attending events perceived as potentially harmful.

From the time my pregnancy was confirmed, my family told me not to move around too much or do any heavy work. They wouldn't let me go out alone after evening. Sometimes it felt restrictive, but I also felt they were caring for me.

#### 3.3.2 ‘*Bhog bharai’* and sacred food preparation

Participants described the *Bhog Bharai ki Rasam* (Ritual of Food Offering) as occurring at different stages following childbirth. In some cases, it was performed in the early postpartum period, once the mother and newborn were considered stable and the initial phase of seclusion had begun to ease. This version of the ritual involved the preparation of two types of rice: plain white rice for household consumption and turmeric-coloured rice, which was specifically prepared as an offering. The turmeric-coloured rice was offered to local household and lineage deities such as Nagnechiya Mata *(Kul Devi)* & other *devatas* (local deities), who were believed to safeguard the household and ensure the well-being of its members. Participants described these deities as protective forces also invoked during other life-cycle events, such as marriage rituals performed when a bride enters her marital home.

The preparation of these offerings was typically undertaken by senior women in the household, particularly mothers-in-law or other elder female relatives, who were regarded as custodians of ritual knowledge. In some instances, younger women assisted under their guidance, reflecting the intergenerational transmission of ritual practices. Some participants described the *Bhog Bharai ki Rasam* as taking place after the completion of six months following childbirth, marking an important stage in the infant's early life. In this form, the prepared turmeric-coloured *bhog* (offering) was taken to local shrines *(devra)*, where it was offered to widely revered local deities, most commonly Baba Ramdev ji in the Bikaner region. Families described placing the offering near or at the mouth of the deity's idol as part of the ritual. In this way, the offering extended from household-level lineage deities *(‘Kul Devi’)* to regional protective deities, linking domestic and community-based systems of belief. Participants also emphasized that the offered food was not to be shared outside the household. As one woman explained:

During Bhog Bharai we make two kinds of rice. The plain one is for the family, and the turmeric rice is for offering. The turmeric rice is offered first. Whatever remains must stay within the house. Giving it outside is strictly avoided. We usually feed it to the animals so that all blessings remain inside.

Following the offering, the food was either consumed within the household or given to domestic animals, and in some cases, a portion was fed to the child. Participants associated these practices with maintaining auspiciousness within the household while seeking blessings for the child's health, protection, and well-being. Families described clear expectations about the movement of ritual food. Leftovers from the offering were kept within the home and not given to outsiders. A participant stated,

Ritual food never goes outside. We give it to the household animals.

According to participants, keeping the food within the household ensured that the blessings associated with the ritual remained contained and undisturbed, while distributing it to domestic animals allowed it to be used without extending it beyond the ritual boundary.

#### 3.3.3 Purifying the mother's milk and first feeding practices

Participants described specific ritual practices performed immediately after childbirth, prior to the initiation of breastfeeding. These practices were considered necessary to ensure that the “first milk” was pure and safe for the newborn. Participants referred to actions such as lighting a lamp *(diya)* and using its ghee (clarified butter) in symbolic purification rituals. In some instances, cotton was dipped in the ghee and touched seven times to a religious image drawn in brown or red colours on the wall of the postnatal ward (Images 1) as well among the women who had to stay in the hospital for more than 24 h. Participants reported that this ritual was performed after discharge upon reaching home, on the household wall in the room where the mother and baby stayed. At home this ritual is performed using red or white colour powder *(Geru)* which is used only in religious rituals and practices. This ritual was followed by a gesture of taking *balaiya*, a protective ritual practice in which a person symbolically wards off negative influences, the evil eye, or harm from another individual, often by moving the hands or ritual objects around the person's body. In this context *Balaiya* over the mother was performed by the elderly women in the family, most commonly the Mother-in-law and husband's elder brother's wife *(Jethani)*, who moved their hands seven times around the woman's body, while holding the ghee-soaked cotton in the hand, which was later burned. Participants explained that this practice was performed to protect the mother from negative influences during the vulnerable period following childbirth. Later, the same ghee soaked cotton was applied to the mother's breast and nipple and massaged gently before initiating breastfeeding.

When the baby is born… the first milk must be purified. When my baby was born, the grandmother did a small ritual and then said, ‘Now the milk is pure, now feed.’ I was scared at first, not knowing what to do, but she explained everything…We follow what our elders taught us. They know what is good for the mother and baby…

Participants described this guidance as something learned from mothers, mothers-in-law, and grandmothers through observation and participation. Rather than being limited to verbal instruction, these practices were learned by watching elder women perform rituals and by following their guidance during the early postpartum period. In addition to these purification practices, early feeding rituals for the newborn often included the administration of small quantities of honey or ghee, typically given by a grandmother or another female elder. Participants described this as the baby's “first taste” before breastfeeding, marking an important moment of introduction to nourishment and social belonging. Families viewed these substances as gentle and strengthening for the newborn.

Here, the grandmother gives a drop of honey or ghee, just one drop. They say the child will speak sweetly and life will be sweet. When my daughter was born, I saw how lovingly everyone offered that one drop… it felt like she truly entered the family.

Participants explained that these practices were typically carried out immediately after birth by elder women, particularly grandmothers or mothers-in-law, who guided the new mother through the early feeding process. When births took place in hospital settings, such practices were sometimes adapted or performed symbolically after returning home.

### 3.4 Postpartum care and support

Participants described the postpartum confinement period, locally known as *Jaapa*, as a central component of maternal recovery and newborn protection from outside world. This phase typically lasted about one month and was marked by rest, restricted movement, and close involvement of female relatives.

#### 3.4.1 Rest and restricted movement

Women explained that remaining indoors during *Jaapa* was considered necessary for physical recovery and for safeguarding the baby from external influences/infections. Leaving the house or performing household work was strongly discouraged. One mother noted,

During Jaapa we do not go out at all. Only rest… it feels like the body has to come together again. At first I would get restless staying in bed, but my mother-in-law would say, ‘Rest, only then you’ll recover.’ And she was right.

Participants described this period as one in which the mother regained strength while adapting to the responsibilities of newborn care.

Support from women kin was described as essential to completing *Jaapa* successfully. The husband's sister (*nanad*), mother-in-law, and sometimes the woman's own mother played key roles. These relatives assisted with daily tasks such as preparing meals, massaging the mother, bathing the newborn, and advising on feeding and rest. As one woman expressed,

My sister-in-law… honestly, without her Jaapa wouldn't even happen. Morning to night she cooked for me, massaged me with oil. Sometimes she would scold me gently…‘Don't eat that’…but even that was out of care.

Participants described this support as easing the mother's workload and providing emotional reassurance during a vulnerable period. Participants' descriptions of Jaapa emphasized a temporary change in the mother's everyday activities. During this period, women remained indoors, reduced household work, and relied more heavily on female relatives for food, physical care, and guidance.

#### 3.4.2 Protective rituals

Alongside physical care, women relatives performed small protective rituals believed to safeguard the mother and baby. These included waving a lit lamp at dawn and dusk, applying soot or turmeric marks on doorways, and keeping the sleeping area warm and quiet. One participant shared,

Every evening my sister-in-law would bring a lamp and say, ‘Come, let's ward off the evil eye.’ She would move the lamp around me and the baby. Sometimes I would laugh, but she said, ‘Laugh if you want, but this is important.’ It also lightened the atmosphere in the house.

These rituals were understood as creating a protective environment around the household, reinforcing feelings of safety and belonging. Women described these practices as part of the everyday care provided during Jaapa, with elder female relatives taking responsibility for performing the rituals and explaining their importance to younger women.

Section III: Incorporation

### 3.5 Infant cleansing and protection rituals

Participants described a series of early-life rituals intended to cleanse, protect, and gradually integrate the newborn into the social and spiritual world of the household. These practices were performed within the first weeks and months of life and were seen as essential for ensuring the infant's well-being.

A commonly described practice was cleaning the body hair of the newborn, in which the newborn's face and body were gently massaged with a soft ball of dough. Women explained that this ritual removed “heaviness” and offered protection from unwanted influences. One participant shared,

The baby kept crying… so the grandmother said, She made a small dough ball, applied ghee, and gently rolled it over the baby's face and body. The baby would quiet down and we felt as if something heavy had been lifted.

This ritual was described as both cleansing and soothing for the infant, often performed by a grandmother or another experienced female elder. Participants described the involvement of grandmothers and other elder women as an important source of guidance during these early-life practices. Younger mothers often learned the practices by observing how elder women performed them.

On the sixth day after birth, families observed *Chathiar*, a small purification ceremony marking the infant's early transition into the household. Participants described activities such as lighting a lamp, making a protective mark with *kajal* on the baby's forehead, and offering prayers. One grandmother explained,

On the sixth day we do the same things every time…we light a lamp, apply kajal, and perform a small ritual. That day I felt the baby truly became part of the household. My mother-in-law said, ‘From today the baby's life begins.

Women noted that *Chathiar* affirmed both spiritual protection and the family's collective welcome to the newborn into the household. Participants particularly associated Chathiar with the newborn's increasing acceptance within the household. The ceremony was described as a point at which the baby was more openly recognized as a member of the family and included within household rituals and practices.

Another widely practiced ritual involved sprinkling *Ganga Jal* (Holy water from the Ganges) around the sleeping area and throughout the house. Participants viewed this as purifying the environment and ensuring a clean, positive and auspicious space for the newborn. Some also applied a drop of the water to the baby's forehead or chest as a blessing. Families described these actions as maintaining a sacred environment during the early days of life.

We consider Ganga water very sacred. I would see my mother-in-law sprinkle it around the room, near the bed, the cradle. She would say, ‘It purifies the air.’ It gave me a strange sense of calm too.

### 3.6 ‘*Annaparashan’*: introducing first solid food

Participants described several rituals that marked key developmental milestones in the infant's early life and signaled their gradual incorporation into family and social life. One such ritual was *Annaprashan*, performed at around six months, marking the infant's first intake of solid food, typically rice. This event was described as an important moment indicating the child's growth and transition beyond exclusive breastfeeding.

One participant stated:

After six months, when we fed rice for the first time… everyone got ready as if it was a wedding. They gave one small spoonful, and that single spoon felt so full of love that I started crying. I felt, ‘now my baby is growing’.

Women explained that the ritual often included prayers, feeding by an elder family member, and a small gathering of relatives. Participants described this event as both a nutritional transition and a symbolic moment of inclusion, where the child was gradually introduced into shared family practices and social life. Families viewed these substances as gentle, auspicious, and strengthening for the newborn, marking an important first moment of nourishment.

## 4 Discussion

This study demonstrates that pregnancy, childbirth, and the postpartum period are socially organized transitions structured through ritual practices, kinship relations, and systems of care. Drawing on rites of passage theory, the findings suggest that reproductive transitions are not experienced solely as biological processes but are collectively managed through culturally meaningful practices that guide women and newborns through stages of separation, transition, and incorporation. Rituals surrounding pregnancy, childbirth, and infant care provide social recognition, emotional reassurance, and protection during periods of uncertainty while simultaneously reinforcing family relationships and lineage continuity.

These rituals collectively structure pregnancy and childbirth as a rite of passage. Godbharai marks public recognition of pregnancy and the woman's transition toward motherhood; postpartum confinement represents a liminal period characterised by vulnerability and separation from ordinary social life; and rituals such as *Chathiar* and *Annaprashan* facilitate the incorporation of mother and child into family and community life. In particular, these rituals help bring the mother and newborn into the husband's family, strengthening relationships within the household and marking the child's place in the lineage. Through the participation of elder women and extended family members such as grandparents, aunts, uncles, mothers-in-law, and sisters-in-law, these events strengthen family roles and support systems. Similar research has shown that such support systems can reduce stress and improve emotional well-being during pregnancy (21).

In the present study, participants described pregnancy as a “delicate” period requiring careful attention to diet, rest, and daily activities in order to protect both the mother and the unborn child. Women reported following culturally guided dietary practices and activity restrictions, which were understood as essential for maintaining maternal health and preventing complications. Studies from different parts of India show similar patterns, where families encourage pregnant women to rest more, stay indoors, and avoid “harmful influences” to prevent complications (22–25). Such practices reflect broader cultural frameworks in which reproduction is not only biological but also deeply embedded within social structures, kinship relations, and normative expectations that regulate women's roles and behavior within the household (26). Rather than being seen solely as restrictive, these practices are often understood by participants as forms of care and protection, shaped by intergenerational knowledge and family support systems.

These practices may also be interpreted through Turner's (1969) concept of liminality. Pregnancy and the postpartum period were consistently described by participants as delicate and vulnerable phases requiring heightened protection and supervision. Dietary restrictions, activity limitations, and postpartum confinement can therefore be understood not merely as health-related behaviours but as symbolic mechanisms through which families manage uncertainty during reproductive transitions. By providing structure, guidance, and protection, these practices help women navigate a period characterized by physical, emotional, and social transformation (17, 18).

These practices create a sense of safety and comfort, helping women feel cared during a physically and emotionally demanding time. However, research also shows that some restrictions, such as avoiding nutritious foods or reducing movement too much, can have negative effects on health, including weakness, poor weight gain, or, in severe cases, risks for the newborn (23). Even so, families continue these practices because they are understood to protect the mother and child. Our informants confirmed that experiencing these practices help women feel supported and give meaning to the many changes experienced during pregnancy and the postpartum period, including recovery after childbirth, initiation of breastfeeding, and adjustment to caring for the newborn. Such practices can also be understood within broader social contexts in which women's behavior during pregnancy and after childbirth is shaped by family norms, expectations, and hierarchies, particularly within the marital household (26).

The study also highlights the central role of elder women in the household. Grandmothers, mothers-in-law, and other elder female relatives, such as *chachi* (father's younger brother's wife), *bua* (father's sister), *nanad* (husband's sister) and *mausi* (mother's sister), are seen as guides who teach the younger women how to behave, what to eat, and how to take care of themselves. They share their own stories and explain the purpose of different rituals. This is an example of “social learning,” where people learn by observing and listening to those around them (27). Because of this guidance, younger women often feel calmer and more confident, even when they are worried about childbirth or early childcare. However, access to such support is not uniform; women who are geographically distant from their natal or affinal kin, or who reside in nuclear households, may experience gaps in guidance, care, and emotional support during pregnancy and the postpartum period (19, 20, 28). In such contexts, the absence of elder women's involvement can increase uncertainty and vulnerability, highlighting how women's reproductive experiences remain closely shaped by the availability of kin-based support within the household structure (26).

At the same time, the findings show that some long-held practices may have negative effects on health. For example, strict food restrictions during pregnancy and after childbirth can reduce important nutrients such as iron, protein, calcium, and folic acid, which pregnant and breastfeeding women need to stay healthy. Avoiding sunlight or fresh air during postpartum confinement may also weaken the body and affect mental well-being. In some families, ritual rules, such as waiting for the *Chathiar* ceremony before taking a newborn out of the house, or delaying breastfeeding until certain prayers are completed, can postpone timely feeding or medical help. Studies from other parts of India show that delaying colostrum feeding, avoiding early skin-to-skin contact, or relying only on home remedies during newborn illness can increase the risk of infections and, in severe cases, even lead to neonatal complications (29). These findings remind us that while traditions bring emotional comfort, they must be followed with care so that the mother's and baby's health is not put at risk. The findings also illustrate how biomedical settings can modify the way these practices are performed rather than simply replacing them. Participants described adapting some rituals when childbirth or postpartum care took place in hospital settings. For example, practices related to purification, protection, and the initiation of breastfeeding were sometimes performed symbolically in the hospital or completed after the mother and newborn returned home. Similarly, participants described negotiating ritual expectations with the practical requirements of hospital care, particularly when rituals involved specific timing, materials, or the presence of elder female relatives. In these situations, families did not necessarily abandon the practice; instead, they modified its timing, location, or form so that it could be continued alongside biomedical care. The interaction between hospital protocols and family expectations was particularly evident in practices surrounding the placenta and early breastfeeding. Hospital staff approached the placenta according to biomedical procedures, whereas some families viewed it as an important part of the newborn that required specific ritual handling and burial. This difference in understanding could create moments of negotiation between clinical procedures and family expectations. Similarly, although hospital care encouraged timely initiation of breastfeeding, some families retained practices involving purification of the first milk or the administration of a small amount of honey or ghee before breastfeeding. Participants' accounts suggest that families responded to these differences by modifying when and where rituals were performed, rather than necessarily abandoning them. Such adaptations allowed families to maintain practices that were meaningful to them while accommodating the requirements of institutional care.

Another important finding relates to how families in Bikaner treat the placenta after birth. Many women and elders explained that the placenta is not medical waste but something connected to the baby. Because of this belief, families often bury the placenta carefully in a clean place near the home or inside the courtyard, sometimes adding turmeric, ash, or salt to protect it from harm. Such practices are meant to safeguard the baby's health and future, especially their strength and long life. From the perspective of rites of passage theory, placental burial may also be understood as an act of incorporation. Participants repeatedly described the placenta as remaining connected to the child even after birth, suggesting that its careful handling forms part of the newborn's transition into social life. By burying the placenta within the household compound, families symbolically anchor the child within domestic space, kinship networks, and lineage structures. In this sense, placental rituals extend the process of incorporation beyond birth itself and reinforce the child's belonging within the family (2, 3, 5, 9, 30).

These practices can also be understood within broader cultural ideas of bodily substance and relatedness, where elements such as the placenta are seen as carrying a continuing connection between the child, the mother, and the household, and where their placement within the domestic space helps establish this relationship (20, 28). Similar rituals have been documented in many parts of India and the world, where the placenta is treated with respect because it is believed to hold spiritual or emotional ties to the newborn (21, 22). These actions help families feel that they are giving extra protection to the baby even after birth. However, some studies also show that unsafe handling or delaying hospital disposal due to ritual expectations may create hygiene risks or delay attention to postpartum needs (31). This means that while these customs are socially significant, guidance may be helpful to ensure safety for both mother and child.

Another important finding is that younger women's views and practices are changing through greater exposure to formal education, health services, and biomedical advice. Participants described questioning some practices while continuing others that remained meaningful to them and their families. Rather than completely rejecting customary practices, some women attempted to combine biomedical recommendations with family traditions. This blending of new ideas and established customs can be understood as a form of cultural adaptation, through which families retain culturally meaningful practices while adjusting them in response to new information and changing circumstances (32). Similar adaptations were evident in hospital settings, where rituals that could not be performed in their usual form were sometimes delayed, modified, or completed after returning home. This suggests that contact with biomedical institutions does not necessarily result in the disappearance of customary practices; instead, families may selectively modify rituals according to the circumstances of childbirth and postpartum care. Such adaptations illustrate how reproductive traditions can be maintained while changing in response to new knowledge, institutional requirements, and women's own experiences of healthcare.

Taken together, these findings suggest that pregnancy rituals in Rajasthan constitute more than a collection of cultural customs. They form an interconnected system through which reproductive transitions are organized, uncertainty is managed, and kinship relations are reproduced. Through ceremonies such as *Godbharai*, postpartum confinement practices, placental rituals, *Chathiar*, and *Annaprashan*, women and newborns are progressively recognized, protected, and incorporated into family and lineage structures. Viewed through the combined lenses of rites of passage, liminality, and social reproduction, these practices reveal how reproduction is simultaneously biological, social, and relational. At the same time, participants' narratives demonstrate that rituals are also expressions of care, protection, hope, and collective responsibility. Understanding these meanings can help healthcare providers build trust with families and develop culturally sensitive maternal and newborn health interventions. Rather than treating ritual practices and biomedical care as opposing systems, these findings suggest the importance of recognizing how families negotiate between the two. Some rituals may be modified in response to hospital protocols, delayed until the family returns home, or performed in attenuated forms that maintain their perceived cultural significance. Maternal and newborn health programs may therefore be more effective when healthcare providers acknowledge the social and emotional meanings attached to these practices while working with families to ensure that adaptations remain compatible with safe and evidence-based care (19, 20, 28).

Based on these findings, we recommend that maternal and newborn care incorporate greater attention to culturally important practices. During routine antenatal and postnatal counselling, health workers can ask women and families about practices related to diet, breastfeeding, postpartum care, and newborn rituals. With the woman's consent, elder female relatives can be included in counselling, particularly where they influence household decisions. Providers can discuss in advance which practices can be continued without affecting essential care, while clearly explaining when a practice may interfere with timely breastfeeding, infection prevention, or newborn safety.

### 4.1 Limitations and transferability

This study has several limitations. The sample was relatively small and drawn from a single district in Rajasthan, with participants predominantly from Hindu communities; therefore, the findings may not be transferable to other regions or religious communities with different cultural and reproductive practices. The study was also conducted primarily in a rural setting, and the experiences of women living in urban or more diverse social environments may differ. Although the study included participants with varying ages and reproductive experiences, the sample was not designed to represent different educational or socioeconomic groups. In addition, many participants had secondary or higher levels of education, which may limit the transferability of the findings to women with lower levels of formal education or different socioeconomic circumstances. Some accounts, particularly those relating to past events or practices that had already occurred, may also have been influenced by participants' recall and personal interpretations. Although in-depth interviews were complemented by participant observation, these factors should be considered when interpreting the findings. Further ethnographic research across different regions, religious communities, urban and rural settings, and diverse educational and socioeconomic groups would help assess how these reproductive rituals and their meanings vary across contexts.

## 5 Conclusion

The findings show that rituals, advice from elder women, and shared beliefs about protection play an important role in how women experience this period of their lives. These practices give women emotional support, a sense of safety, and a feeling of belonging within their families. At the same time, they also function as important social processes through which women and their newborns are publicly recognized and incorporated into the husband's family and lineage. Through these collective practices, relationships within the household are affirmed, and the position of the mother and child within the family structure is strengthened. At the same time, the study found that some practices, especially strict food rules, confinement, or delays in seeking medical help, may have negative effects on the health of mothers and newborns if followed without caution. This study suggest that cultural practices around pregnancy are meaningful and valuable, but they also need to be balanced with safe pregnancy care guidelines and health behaviours. Understanding both the positive and risky aspects of these traditions can help families, healthcare professionals, and community leaders support women in healthier and more respectful ways.

## Data Availability

The original contributions presented in the study are included in the article/Supplementary Material, further inquiries can be directed to the corresponding author.

## References

[1] Eunice Kennedy Shriver National Institute of Child Health and Human Development - NICHD. Available online at: https://www.nichd.nih.gov/health/topics/factsheets/pregnancy (Accessed June 21, 2026).

[2] Davidson JR. The shadow of life: psychosocial explanations for placenta rituals. Cult Med Psychiatry. (1985) 9(1):75–92. 10.1007/BF00048538 2580666

[3] Moeti C Mulaudzi FM Rasweswe MM. The disposal of placenta among Indigenous groups globally: an integrative literature review. Int J Reprod Med. (2023) 2023:1–10. 10.1155/2023/6676809 PMC10622600 37927303

[4] Cultural perspectives on the placenta. Case Rep.

[5] Human maternal placentophagy: a survey of self-reported motivations and experiences associated with placenta consumption - PubMed. Available online at: https://pubmed.ncbi.nlm.nih.gov/23445390/ (Accessed June 21, 2026). 10.1080/03670244.2012.719356 23445390

[6] Placentophagy: self-trust and embodied entanglements in Czech society | Medical Humanities. Available online at: https://mh.bmj.com/content/early/2026/03/17/medhum-2025-013718.abstract (Accessed June 21, 2026). 10.1136/medhum-2025-013718 41844269

[7] Consumption of Maternal Placenta in Humans and Nonhuman Mammals: Beneficial and Adverse Effects. Available online at: https://www.mdpi.com/2076-2615/10/12/2398 (Accessed June 21, 2026). 10.3390/ani10122398 PMC7765311 33333890

[8] Thieme E-Journals - Geburtshilfe und Frauenheilkunde/Full Text. Available online at: https://www.thieme-connect.com/products/ejournals/html/10.1055/a-0674-6275 (Accessed June 21, 2026).

[9] Choudhary R Gothwal S Nayan S Meena BS. Common ritualistic myths during pregnancy in northern India. Int J Contemp Pediatr. (2017) 4(5):1644. 10.18203/2349-3291.ijcp20173628

[10] Gatrad AR Ray M Sheikh A. Hindu birth customs. Arch Dis Child. (2004) 89(12):1094–7. 10.1136/adc.2004.050591 15557039 PMC1719756

[11] Choudhry UK. Traditional practices of women from India: pregnancy, childbirth, and newborn care. J Obstet Gynecol Neonatal Nurs. (1997) 26(5):533–9. 10.1111/j.1552-6909.1997.tb02156.x 9313183

[12] Sethi GK Saini NK. Prevalence of superstitions in Indian society in 21st century. Int J Nurs Educ. (2019) 11(4):56–60. 10.5958/0974-9357.2019.00089.8

[13] Hagaman A Gonzalez Rodriguez H Egger E Bitewulign B Case H Alemayehu AK. Navigating and manipulating childbirth services in afar, Ethiopia: a qualitative study of cultural safety in the birthing room. Soc Sci Med. (2023) 331:116073. 10.1016/j.socscimed.2023.116073 37481879 PMC10410251

[14] Sihag R Tyagi S Shinde KP. Preserving indigenous livestock wisdom: A study of rural women’s goat rearing practices in Bikaner.

[15] Gupta V Saxena R Walia GK Agarwal T Vats H Dunn W. Gestational route to healthy birth (GaRBH): protocol for an Indian prospective cohort study. BMJ Open. (2019) 9(4):e025395. 10.1136/bmjopen-2018-025395 31048433 PMC6501957

[16] Nowell LS Norris JM White DE Moules NJ. Thematic Analysis. (2017). Available online at: https://journals.sagepub.com/doi/10.1177/1609406917733847 (Accessed June 21, 2026).

[17] Van Gennep A. The Rites of Passage. Chicago: University of Chicago Press (1909). References - Scientific Research Publishing. Available online at: https://www.scirp.org/reference/referencespapers?referenceid=1545080 (Accessed June 21, 2026).

[18] Turner V. The Ritual Process. Structure and Anti-Structure. Brunswick and London: Aldine Transaction (1969). References - Scientific Research Publishing. Available online at: https://www.scirp.org/reference/referencespapers?referenceid=2104211 (Accessed June 21, 2026).

[19] Carsten J. After Kinship. Repr. Cambridge: Cambridge Univ. Press (2007). p. 216. New departures in anthropology.

[20] Stone L. Cultures of relatedness: new approaches to the study of kinship. Am Ethnol. (2001) 28(3):690–2. 10.1525/ae.2001.28.3.690

[21] Routledge & CRC Press. Birth as an American Rite of Passage. Available online at: https://www.routledge.com/Birth-as-an-American-Rite-of-Passage/Davis-Floyd/p/book/9780367431297 (Accessed June 21, 2026).

[22] Hollen V Coale C. Birth on the Threshold: Childbirth and Modernity in South India. Berkeley: University of California Press (2003). Available online at: https://hdl.handle.net/2027/heb09215.0001.001 (Accessed Jun 21, 2026).

[23] O’Neil S Naeve K Ved R. An Examination of the Maternal Health Quality of Care Landscape in India).

[24] Maurya S Dixit K Attri S Mukherjee R Anand VK Mazumder S. Exploring positive deviant behaviors among high-risk pregnant women with different birth outcomes: a qualitative study from Palwal, India. Cureus. (2025) 17(9):e91584. 10.7759/cureus.91584 41049916 PMC12495533

[25] Multifaceted Determinants of Breastfeeding Practices in Udaipur, Rajasthan | Indian Journal of Community Health. Available online at: https://www.iapsmupuk.org/journal/index.php/IJCH/article/view/2945 (Accessed June 21, 2026).

[26] Patel T. Fertility Behaviour: Population and Society in a Rajasthan Village. Delhi; New York: Oxford University Press (1994). p. 287. Gender studies.

[27] Bandura A Walters RH. Social Learning Theory (Vol. 1). Englewood Cliffs, NJ: Prentice-Hall (1977). References - Scientific Research Publishing. Available online at: https://www.scirp.org/reference/referencespapers?referenceid=2730439 (Accessed June 21, 2026).

[28] Lambert H. Village bodies: constitution, locality and affection in rajasthani kinship. In: Rao A, editor. Concepts of Kinship in South Asian Practice. New York: Berghahn Books (2000). p 81–100.

[29] Raman S Nicholls R Ritchie J Razee H Shafiee S. Eating soup with nails of pig: thematic synthesis of the qualitative literature on cultural practices and beliefs influencing perinatal nutrition in low and middle income countries. BMC Pregnancy Childbirth. (2016) 16(1):192. 10.1186/s12884-016-0991-z 27464710 PMC4964025

[30] Oe F Oa A Ri F Ag A. Postpartum Placental Rituals: Perceptions of Midwives and Mothers in Selected Healthcare Centers, Southwestern Nigeria.

[31] Vernekar PP. A study on traditional beliefs and practices during the postpartum period among mothers at a district hospital in goa. Int J Prev Curative Community Med. (2021) 07(03):1–11. 10.24321/2454.325X.202111

[32] Kant A. Experiencing pregnancy: negotiating cultural and biomedical knowledge. Sociol Bull. (2014) 63(2):247–62. 10.1177/0038022920140204

