# Supplementary material for: “We follow what our elders taught us”: an ethnographic study of kinship, rituals, and reproductive transitions among women in the GaRBH cohort, Rajasthan, India
Source: Front Glob Womens Health. 2026 Sep 30;7:1915333. doi: 10.3389/fgwh.2026.1915333 (PMC13661947; doi:10.3389/fgwh.2026.1915333)
Supplement: Supplementary file 1 [file Datasheet1.docx]

Title: “We Follow What Our Elders Taught Us”: Rituals, Kinship, and Reproductive Transitions among Women in the GaRBH Cohort, Rajasthan, India

Supplementary Material

# 1 Supplementary Tables

**Supplementary Table 1.1 : Emergent Themes and Sub-themes with Illustrative Verbatim Quotes**

| **Themes** | **Sub-themes** | **Verbatim** |
| --- | --- | --- |
| Pregnancy Celebrations and Preparation Rituals | *Mehendi* for Comfort and Blessing | “Jab… jab haath par mehendi lagwati hoon na, toh bas… mann ko ek shanti si milti hai. Jaise thoda thandak aa jaati hai sharir mein bhi. Haan… matlab lagta hai ki ghar mein khushi ka time chal raha hai.”  (“When I get mehendi applied on my hands, I feel a kind of calm. It brings a cooling sensation in the body. It feels like a happy time in the family.”) |
|  | *‘Godbharai’* as Collective Celebration | “Godbharai mein sab… sab milkar god bharte hain…anaaj, mithai, nariyal, peela kapda. Wo sab dete waqt bolte hain ki, ‘ye maa aur bachche ke liye achha hota hai.’ Aur sach mein… uss din lagta hai ki sab mere saath hain, sab dua de rahe hain.”  (“During Godbharai, everyone gathers to fill the lap with grains, sweets, coconut, and yellow cloth. While giving these things, they say it is good for the mother and child. And truly, on that day, it feels like everyone is with me, everyone is giving their blessings.”)  “Rasm hoti hai toh ghar mein ek garmaahat si aa jaati hai. Saas, nanad, sab thoda pyaar se baat karte hain. Aise waqt pe lagta hai ki pregnancy sirf dard aur dikkat nahi hoti… khushi bhi hoti hai.”  (“When a ritual takes place, there is a warmth in the house. My mother-in-law, sister-in-law, everyone speaks more gently. At that time I feel that pregnancy is not only pain and difficulty… there is joy too.”) |
|  | Gifts as Expressions of Care and Support | “Godbharai ke din sab auratein milkar gaati hain, hansati-bolti hain… kabhi mazaak, kabhi purani kahaniyan. Us beech mein main sochti hoon…‘achha, main akeli nahi hoon.’ Par… (pause) kabhi-kabhi mann dukhta bhi hai, jaise jab ghar mein jhagda hota hai ya koi saath dene wala nahi hota. Par jab ye rasmein hoti hain, toh dil ko sahara milta hai.”  (“On the day of Godbharai, all the women sing together, laugh, talk….sometimes jokes, sometimes old stories. In those moments I feel, ‘yes, I’m not alone.’ But… (pause) sometimes it hurts too, like when there is conflict at home or no one to support me. But during these rituals, the heart feels held.”)  “Meri Godbharai toh hui hi nahi… kaun karta? Bas main aur mera pati hi yahan rehte hain, aur wo bhi roz kaam par rehte hain. Kabhi-kabhi lagta hai… (long pause) ki shayad main kisi ko important hi nahi lagti. Jab doosri auraton ki videos dekhti hoon, jahan poora parivaar jama hota hai, toh mann bhar aata hai.”  (“My Godbharai never happened… who would do it? It’s just me and my husband here, and he is out working all day. Sometimes I feel… (long pause) maybe I’m not important to anyone. When I see videos of other women whose whole families gather, I feel emotional.”) |
| Symbolic Meaning and Beliefs Associated with the Placenta |  | “Aawal… wo bas ek hissa nahi hota. Hum log kehte hain na, ‘pehla rakshak,’ toh wohi hai. Jab tak bachcha pet mein rehta hai, wohi sambhalta hai. Mere se ek baar poocha gaya hospital mein ki isko fek dein? Maine turant bola, ‘Nahi, yeh bachche ke saath rehta hai, ise sambhal ke rakhna.’”  (“The placenta is not just a part. We call it the ‘first guardian,’ and that’s what it is. As long as the baby is in the womb, it protects. Once a hospital person asked me if they should throw it away. I immediately said, ‘No, it stays with the baby, keep it safe.”)  “Ye bachche ke saath hi rehta hai, isliye isko sambhal ke rakhna padta hai.”  (“It stays with the baby throughout, so it must be taken care of properly.”) |
|  | Placental Burial Practice within Household Space | “Hum toh rasoi ke kone mein hi daabte hain. Wo jagah… matlab, sabse pavitra hoti hai. Jab main pehli baar maa bani, meri saas ne mujhe pakad ke dikhaya ki ‘yahan daalte hain,’ aur bola ki isse bachche ki kismat ghar mein hi bani rehti hai… bahar nahi jaati.”  (“We always bury it in the corner of the kitchen. That place… I mean, it is the most sacred. When I became a mother for the first time, my mother-in-law held my hand and showed me where they bury it, saying it keeps the baby’s good fortune inside the home.”) |
|  | Ritual Handling of *‘Awal’* After Birth | “Kabhi-kabhi hum log thoda sa hissa chandi mein bandh kar rakh lete hain. Haan… chhota sa. Saas bolti thi, ‘Nazar zyada lagti hai naye bachchon ko, isse bachcha bacha rahta hai.’ Mere do bachche hain, dono ka rakha hua hai mere paas.”  (“Sometimes we wrap a small portion in silver. Yes… just a little bit. My mother-in-law would say, ‘Newborns attract the evil eye, this protects them.’ I have two children, and I still have the pieces for both.”) |
| Food, Purity, and Early Nourishment | Dietary Practices and Activity Restrictions during Pregnancy | “Jab se pata chala ki main pregnant hoon, tab se ghar wale bolte the ki zyada chalna-phirna nahi, bhaari kaam bilkul nahi karna. Shaam ke baad bahar bhi akela nahi bhejte the……Kabhi-kabhi lagta tha zyada rok-tok hai…par andar se lagta tha ki wo meri fikr kar rahe hain.”  (“From the time my pregnancy was confirmed, my family told me not to move around too much or do any heavy work. They wouldn’t let me go out alone after evening. Sometimes it felt restrictive, but I also felt they were caring for me.”)  “Bahut dhyaan rakhna padta hai… har cheez nahi khate. Dahi, papita, ananas jaise cheezein thandi hoti hain, wo mana karte hain ghar wale. Saas bolti hain ki ‘ye sab se bachcha kamzor ho sakta hai.’ Isliye main zyada ghee, doodh, aur panjiri leti thi, taki taqat bani rahe.”  (“Now has to be very careful… you can’t eat everything. Foods like curd, papaya, and pineapple are considered ‘cold,’ so they are avoided. My mother-in-law would say these could make the baby weak. So I mostly consumed ghee, milk, and panjiri to maintain strength”) |
|  | ‘*Bhog Bharai’* and Sacred Food Preparation | “Bhog Bharai mein hum do tarah ka chawal banate hain. Safed ghar ke liye, aur haldi wala bhog ke liye. Haldi wala pehle bhagwan ko chadhate hain. Uske baad jo bhi bachta hai, woh ghar mein hi rehta hai. Bahar dena… bilkul mana hai. Hum log toh jaanwaron ko de dete hain, taki sab shubh andar hi rahe.”  (“During Bhog Bharai we make two kinds of rice. The plain one is for the family, and the turmeric rice is for offering. The turmeric rice is offered first. Whatever remains must stay within the house. Giving it outside is strictly avoided. We usually feed it to the animals so that all blessings remain inside.”)  “Bacha hua khana bahar nahi jaata. Ghar ke jaanwaron ko de dete hain.”  (“Ritual food never goes outside. We give it to the household animals.”) |
|  | Purifying the Mother’s Milk | “Jab bachcha paida hota hai na… toh pehle doodh ko pavitra karna zaroori hota hai. Mera bachcha hua toh dadi ne ek chhota sa pooja jaisa kiya, phir bola, ‘ab doodh shuddh ho gaya, ab pila.’ Main pehle dar rahi thi, ki pata nahi kya karna hai, par unhone sab samjha diya.”  (“When the baby is born… the first milk must be purified. When my baby was born, the grandmother did a small ritual and then said, ‘Now the milk is pure, now feed.’ I was scared at first, not knowing what to do, but she explained everything…We follow what our elders taught us. They know what is good for the mother and baby…. ”) |
|  | First Feeding Practices for the Newborn | “Hamare yahan dadi ya badi maa thoda sa shahad ya ghee chatati hain—bas ek boond. Wo kehte hain ki isse bachcha meetha bolna sikhega, aur zindagi meetha rahega. Jab meri beti hui thi, toh maine dekha ki saare log itne pyaar se wo ek boond dete hain… bas lagta hai ki bachcha parivaar mein aa gaya.”  (“Here, the grandmother gives a drop of honey or ghee—just one drop. They say the child will speak sweetly and life will be sweet. When my daughter was born, I saw how lovingly everyone offered that one drop… it felt like she truly entered the family.”) |
|  | ‘Annaparashan’ Introducing First Solid Food | “Chhe mahine baad jab pehli baar chawal khilaya na… toh sab log tayyar ho gaye jaise shaadi ho. Ek chhota sa chamcha diya, aur bas usi ek chamche mein itna pyaar tha ki main ro padi. Mujhe laga, ‘ab mera bachcha bada ho raha hai.’”  (“After six months, when we fed rice for the first time… everyone got ready as if it was a wedding. They gave one small spoonful, and that single spoon felt so full of love that I started crying. I felt, ‘now my baby is growing.”)  “Hamare yahan dadi ya badi maa thoda sa shahad ya ghee chatati hain, bas ek boond. Wo kehte hain ki isse bachcha meetha bolna sikhega, aur zindagi meetha rahega. Jab meri beti hui thi, toh maine dekha ki saare log itne pyaar se wo ek boond dete hain… bas lagta hai ki bachcha parivaar mein aa gaya.”  (“Here, the grandmother gives a drop of honey or ghee, just one drop. They say the child will speak sweetly and life will be sweet. When my daughter was born, I saw how lovingly everyone offered that one drop… it felt like she truly entered the family.”) |
| Postpartum Care and Support | Rest and Restricted Movement | “Jaapa mein hum bilkul bahar nahi nikalte. Sirf aaram… aisa lagta hai jaise sharir ko dobara se jodna hota hai. Pehle-pehle main bechain ho jaati thi bed pe rehte rehte, par saas bolti thi, ‘Aaram kar, tabhi tu theek hogi.’ Aur wo sahi thi.”  (“During Jaapa we do not go out at all. Only rest… it feels like the body has to come together again. At first I would get restless staying in bed, but my mother-in-law would say, ‘Rest, only then you’ll recover.’ And she was right.”)  “Meri nanad… sach bolun toh, bina uske Jaapa hota hi nahi. Subah-shaam woh hi mere liye khana banati thi, tel malish karti thi. Kabhi kabhi thoda daant bhi deti thi ki ‘aisi cheez mat kha,’ par wo bhi pyaar mein hi hota tha.”  (“My sister-in-law… honestly, without her Jaapa wouldn’t even happen. Morning to night she cooked for me, massaged me with oil. Sometimes she would scold me gently…‘Don’t eat that’…but even that was out of care.”) |
|  | Protective Rituals | “Shaam hote hi nanad diya leke aa jaati thi… ‘chalo, nazar utaar dein.’ Wo diya ko mere aur bachche ke chaaro taraf ghumati thi. Main kabhi-kabhi hans padti thi, par wo bolti thi, ‘Hans le, par ye zaroori hai.’ Usse ghar ka mahaul bhi halka ho jaata tha.”  (“Every evening my sister-in-law would bring a lamp and say, ‘Come, let’s ward off the evil eye.’ She would move the lamp around me and the baby. Sometimes I would laugh, but she said, ‘Laugh if you want, but this is important.’ It also lightened the atmosphere in the house.”) |
| Infant Cleansing and Protection Rituals | Dough-Based Cleansing | “Bachcha rote rehta tha… toh dadi ne bola tha…Wo aate ki ek chhoti si loi banati thi, ghee lagati thi, phir usse chehre aur shareer par gol-gol ghumaati thi. Bachcha bhi chup ho jata tha aur hum sab ko lagta tha ki jaise koi bojh utaar gaya.”  (“The baby kept crying… so the grandmother said, She made a small dough ball, applied ghee, and gently rolled it over the baby’s face and body. The baby would quiet down and we felt as if something heavy had been lifted.”) |
|  | ‘Chathiar’ Purification ceremony | “Chhatti wale din hum lambe time se ek hi cheez karte hain…deepak jalate hain, kajal lagate hain, thodi si pooja karte hain. Us din mujhe lagta hai ki bachcha sach mein ghar ka hissa ban gaya…. Saas ne bola tha, ‘Aaj se iski zindagi shuru hoti hai.’”  (“On the sixth day we do the same things every time…we light a lamp, apply kajal, and perform a small ritual. That day I felt the baby truly became part of the household. My mother-in-law said, ‘From today the baby’s life begins.’”) |
|  | Use Holy-Water of River Ganga for purification | “Ganga Jal ko hum bahut pavitra maante hain. Main dekhti thi ki saas chhote chhote chhidke karti thi kamre mein… bed ke paas, palne ke paas. Bolti thi ki ‘isse hawa saaf hoti hai.’ Mere mann ko bhi ajeeb si shanti milti thi.”  (“We consider Ganga water very sacred. I would see my mother-in-law sprinkle it around the room, near the bed, the cradle. She would say, ‘It purifies the air.’ It gave me a strange sense of calm too.”) |

*Both transcribed & Translated verbatim has been provided in “--” & (--) respectively*

**Supplementary Table 1.2: COREQ (COnsolidated criteria for REporting Qualitative research) Checklist**

| ,**Topic** | **Item No.** | **Guide Questions/Description** | **Reported on**  **Page No.** |
| --- | --- | --- | --- |
| **Domain 1: Research team and reflexivity** | | | |
| *Personal characteristics* | | | |
| Interviewer/facilitator | 1 | Which author/s conducted the interview or focus group? | 7 |
| Credentials | 2 | What were the researcher’s credentials? E.g. PhD, MD | 7 |
| Occupation | 3 | What was their occupation at the time of the study? | 7 |
| Gender | 4 | Was the researcher male or female? | 7 |
| Experience and training | 5 | What experience or training did the researcher have? | 7 |
| **Relationship with participants** | | | |
| Relationship established | 6 | Was a relationship established prior to study commencement? | NR |
| Participant knowledge of  the interviewer | 7 | What did the participants know about the researcher? e.g. personal  goals, reasons for doing the research | NR |
| Interviewer characteristics | 8 | What characteristics were reported about the inter viewer/facilitator?  e.g. Bias, assumptions, reasons and interests in the research topic | 7 |
| **Domain 2: Study design** | | | |
| *Theoretical framework* | | | |
| Methodological orientation and Theory | 9 | What methodological orientation was stated to underpin the study? e.g. grounded theory, discourse analysis, ethnography, phenomenology,  content analysis | 7-9 |
| *Participant selection* | | | |
| Sampling | 10 | How were participants selected? e.g. purposive, convenience,  consecutive, snowball | 4-5 |
| Method of approach | 11 | How were participants approached? e.g. face-to-face, telephone, mail,  Email | 4-5 |
| Sample size | 12 | How many participants were in the study? | 5 |
| Non-participation | 13 | How many people refused to participate or dropped out? Reasons? | 4-5 |
| *Setting* | | | |
| Setting of data collection | 14 | Where was the data collected? e.g. home, clinic, workplace | 4,7 |
| Presence of non-  participants | 15 | Was anyone else present besides the participants and researchers? | NR |
| Description of sample | 16 | What are the important characteristics of the sample? e.g. demographic  data, date | 5, 10-11 |
| *Data collection* | | | |
| Interview guide | 17 | Were questions, prompts, guides provided by the authors? Was it pilot  tested? | 7 |
| Repeat interviews | 18 | Were repeat interviews carried out? If yes, how many? | NR |
| Audio/visual recording | 19 | Did the research use audio or visual recording to collect the data? | 7 |
| Field notes | 20 | Were field notes made during and/or after the interview or focus group? | 7 |
| Duration | 21 | What was the duration of the interviews or focus group? | 7 |
| Data saturation | 22 | Was data saturation discussed? | 8 |
| Transcripts returned | 23 | Were transcripts returned to participants for comment and/or | NR |

| **Topic** | **Item No.** | **Guide Questions/Description** | **Reported on**  **Page No.** |
| --- | --- | --- | --- |
|  |  | correction? |  |
| **Domain 3: analysis and Findings** | | | |
| *Data analysis* | | | |
| Number of data coders | 24 | How many data coders coded the data? | 8 |
| Description of the coding  tree | 25 | Did the authors describe the coding tree? | 8 |
| Derivation of themes | 26 | Were themes identified in advance or derived from the data? | 8 |
| Software | 27 | What software, if applicable, was used to manage the data? | 8 |
| Participant checking | 28 | Did participants provide feedback on the findings? | 8 |
| *Reporting* | | | |
| Quotations presented | 29 | Were participant quotations presented to illustrate the themes/findings?  Was each quotation identified? e.g. participant number | 12-20 |
| Data and findings consistent | 30 | Was there consistency between the data presented and the findings? | 10-20 |
| Clarity of major themes | 31 | Were major themes clearly presented in the findings? | 10-20 |
| Clarity of minor themes | 32 | Is there a description of diverse cases or a discussion of minor themes? | 11-20 |
